# Safety and Immunogenicity of a Rederived, Live-Attenuated Dengue Virus Vaccine in Healthy Adults Living in Thailand: A Randomized Trial

**DOI:** 10.4269/ajtmh.13-0452

**Published:** 2014-07-02

**Authors:** Veerachai Watanaveeradej, Robert V. Gibbons, Sriluck Simasathien, Ananda Nisalak, Richard G. Jarman, Angkool Kerdpanich, Elodie Tournay, Rafael De La Barrerra, Francis Dessy, Jean-François Toussaint, Kenneth H. Eckels, Stephen J. Thomas, Bruce L. Innis

**Affiliations:** Department of Pediatrics, Phramongkutklao Hospital (PMK), Bangkok, Thailand; US Army Medical Component–Armed Forces Research Institute of Medical Sciences, Bangkok, Thailand; Viral Diseases Branch, Walter Reed Army Institute of Research, Silver Spring, Maryland; GlaxoSmithKline Vaccines, Wavre, Belgium; Bioproduction Facility, Translational Medicine Branch, Walter Reed Army Institute of Research, Silver Spring, Maryland; GlaxoSmithKline Vaccines, King of Prussia, Pennsylvania

## Abstract

Safety and immunogenicity of two formulations of a live-attenuated tetravalent dengue virus (TDEN) vaccine produced using rederived master seeds from a precursor vaccine were tested against a placebo control in a phase II, randomized, double blind trial (NCT00370682). Two doses were administered 6 months apart to 120 healthy, predominantly flavivirus-primed adults (87.5% and 97.5% in the two vaccine groups and 92.5% in the placebo group). Symptoms and signs reported after vaccination were mild to moderate and transient. There were no vaccine-related serious adverse events or dengue cases reported. Asymptomatic, low-level viremia (dengue virus type 2 [DENV-2], DENV-3, or DENV-4) was detected in 5 of 80 vaccine recipients. One placebo recipient developed a subclinical natural DENV-1 infection. All flavivirus-unprimed subjects and at least 97.1% of flavivirus-primed subjects were seropositive to antibodies against all four DENV types 1 and 3 months post-TDEN dose 2. The TDEN vaccine was immunogenic with an acceptable safety profile in flavivirus-primed adults.

## Introduction

Dengue is an arboviral infection causing a mild to moderate febrile illness with headache, myalgia, rash, and cytopenias. Occasionally, disease becomes severe, with plasma leakage, hemorrhage, and intravascular volume depletion leading to shock and potentially, death.^1^ An estimated 2.5 billion people in over 120 dengue-endemic countries worldwide are at risk of dengue infection, but many believe that this number is an underestimation.^1^

Thailand is endemic for all four dengue virus (DENV) types (DENV-1, DENV-2, DENV-3, and DENV-4), with one or two types predominating at any given time.^2^ In 2006, 23% of the reported dengue cases in the World Health Organization (WHO) Southeast Asia Regional Office (SEAR) were from Thailand.^3^ Comparison of prospective cohort study data with locally reported national surveillance data indicated that dengue incidence was underrecognized by 8.7-fold and that hospitalized dengue cases were underrecognized by 2.6-fold.^4^ The data show that a median 229,886 dengue cases occurred annually from 2003 to 2007, and in 2007, > 95,000 Thai children were estimated to be hospitalized because of dengue.^4^

The Walter Reed Army Institute of Research (WRAIR) has collaborated with GlaxoSmithKline (GSK) Vaccines to develop a live tetravalent dengue virus vaccine candidate attenuated by serial passage in primary dog kidney (PDK) cells.^5^,^6^ After identifying a safe, well-tolerated, and immunogenic formulation of the DENV vaccine candidate in a phase II trial,^7^ two phase I/II clinical trials evaluated the vaccine in flavivirus-naïve Thai subjects given two doses 6 months apart. The first trial was an open label study of seven Thai children.^8^ The second trial was a randomized study of 51 Thai infants/toddlers 12–15 months of age.^9^ Both studies confirmed the acceptable safety profile of the vaccine. Antibody responses to all four DENV types were reported in more than one-half of the infants/toddlers and all of the children 1 month after the second dose. These studies used lyophilized monovalent vaccines that were combined into a tetravalent preparation at the time of administration.

After these studies, a new candidate vaccine was prepared from rederived vaccine strains using the same manufacturing process, with the following exceptions: each strain had three additional passages in fetal rhesus lung (FRhL) cells, monovalent bulks were formulated with a carbohydrate stabilizer rather than human serum albumin, and the final live-attenuated dengue vaccine was lyophilized as a tetravalent product (referred to as the TDEN vaccine). This rederived vaccine has been evaluated in mostly flavivirus-naïve adults in the United States.^10^ In this article, we report the first clinical evaluation of the safety and immunogenicity of the rederived TDEN vaccine in a flavivirus-primed adult population in Thailand.

## Materials and Methods

### Study design.

This study was a phase II, randomized, double blind, controlled trial designed to evaluate the safety and immunogenicity of two doses of the TDEN vaccine administered 6 months apart (Clinicaltrials.gov NCT00370682).

The clinical trial was conducted at the Phramongkutklao Hospital in Bangkok, Thailand from April of 2007 to February of 2008 in accordance with the provisions of the Declaration of Helsinki, good clinical practice, and Thai and US regulations. The clinical protocol and supporting documents were approved by the ethical review committees of the Royal Thai Army, the Thai Ministry of Public Health, and the US Army Office of the Surgeon General's US Army Human Subjects Research Review Board. Written informed consent was obtained from each subject before the performance of any study-specific procedures.

### Role of the sponsor and development partners.

The study was designed by the US Army and GSK Biologicals SA. The study sponsor (US Army Medical Materiel Development Activity [USAMMDA]) and GSK Biologicals SA monitored and reported on subject safety. Investigators collected and encoded the data into a GSK database, and a GSK statistician analyzed the data according to a pre-specified and mutually approved plan. The study was jointly funded by the US Army Medical Research and Materiel Command and GSK Biologicals SA.

### Vaccines.

Two candidate TDEN vaccine formulations, F17 and F19, were tested. Development of these candidate vaccines, including DENV strains, PDK and FRhL cell culture passage, and viral concentration, has been described previously.^10^

The TDEN vaccines in this study were formulated to have comparable *in vitro* potency, except that F19 was planned to contain approximately 10-fold less DENV-4 than F17. *In vitro* potency tests for the vaccines are shown in Table 1. Note that the actual measured difference in F19 DENV-4 potency was fourfold less (the test error was ± twofold).

The F17 and F19 TDEN vaccines post-transfection formulations were pre-mixed, freeze-dried, and presented in single dose vials, with sterile water used to reconstitute the vaccine before injection. The volume of each dose was 0.5 mL. DENV vaccine potency was confirmed using an immunofocus assay (IFA).^8^

The placebo, which seemed identical to the vaccines, contained a cell culture medium (Eagle's Minimum Essential Medium [EMEM]) and was formulated in the same way as the vaccines. Placebo injection volume was the same as vaccine (0.5 mL).

Two doses of vaccine or placebo were given 6 months apart. All doses were administered subcutaneously in the deltoid area.

### Study subjects.

Healthy male and female subjects between 20 and 25 years of age were provided with a detailed explanation of the study and enrolled after an informed consent process. Subjects were in good general health as determined by eligibility screening, including a medical history and a physical examination. Subjects were excluded from the study if positive for hepatitis B surface antigen (HBsAg), hepatitis C virus (HCV), or human immunodeficiency virus (HIV). Complete blood count (CBC), alanine aminotransferase (ALT), and aspartate aminotransferase (AST) levels were also assessed. Clinically significant laboratory abnormalities at screening, receipt of immune-modifying drugs within 90 days of enrollment, and a history of chronic disease were exclusion criteria. Female subjects had to be of non-childbearing potential; otherwise, they must have been either abstinent or using adequate contraceptive precautions from 30 days before vaccination until 60 days after completion of the vaccination series, with a negative pregnancy test taken within 48 hours before vaccination.

Serology for DENV and Japanese encephalitis virus (JEV) was determined after enrollment based on blood samples collected before the first dose was administered.

In total, 120 subjects were enrolled in the trial and randomized into three treatment groups (F17, F19, and placebo) using a block 1:1:1 randomization list from a standard Statistical Analysis System (SAS) program (SAS Institute Inc., Cary, NC). The subjects, investigators, sponsor, GSK Vaccines, and study personnel remained unaware of the treatment assignments until the time of official unblinding after study completion. Subjects were required to attend 11 study visits over a 9-month study period (from day 0 to 2 months after dose 2).

### Safety evaluation.

Safety follow-up occurred throughout the entire study period, including a 21-day follow-up for solicited symptoms, a 31-day follow-up for unsolicited symptoms, and follow-up for the entire study period (day 0 through month 9) for serious adverse events (SAEs) and fever for suspected dengue. Solicited adverse events (AEs) were collected from all subjects using a diary card to record information for 21 days after each dose. Solicited injection site reactions included injection site pain, redness, and swelling. Solicited general symptoms include fever, fatigue, headache, pain behind the eyes, abdominal pain, nausea, vomiting, muscle ache, joint ache, diffuse rash on the trunk, photophobia, and pruritis. To monitor fever, subjects were also provided with digital thermometers to measure and record their oral temperature each evening for 21 days starting on the day of vaccination. Intensities of each AE were scored as grades 1–3, with grade 3 fever defined as an oral body temperature > 39°C (> 102.2°F), grade 3 redness and swelling defined as > 20-mm diameter around the injection site, and all other grade 3 AEs defined as those AEs preventing normal daily activity.

Unsolicited AEs were recorded by subjects over a 31-day follow-up period after each dose. Unsolicited AEs were coded with the use of the *Medical Dictionary for Regulatory Activities*.^11^ SAEs were defined as medically significant events, including those AEs resulting in hospitalization, disability, or death. SAEs were recorded throughout the entire study period.

Throughout the study, the investigators also conducted active surveillance for AEs of cardiac origin. This evaluation was designed to address the theoretical potential for a live DENV vaccine to cause AEs similar to the atypical cardiac manifestations reported after natural DENV infections.^12^ Cardiac-directed medical history and physical examinations focused on the presence of chest pain syndromes, palpitations and arrhythmias, shortness of breath, and syncope or near-syncope. If evidence of cardiac-related signs or symptoms was found during examination, the investigator performed an electrocardiogram (ECG) and blood test for troponin T level.

During the 31-day follow-up period after each dose and during acute illness for suspected dengue, subjects underwent a physical examination that focused on signs and symptoms of dengue, including assessments for skin, mucosal, or conjunctival hemorrhages, conjunctival injection, lymphadenopathy, skin rash, and presence of hepatomegaly or splenomegaly.

In addition to recording body temperatures 21 days after each vaccination, subjects were to consult the investigator if they developed a fever at any time during the study period. Dengue was suspected if a subject had a fever > 39°C (> 102.2°F) at any time or a fever ≥ 38°C (100.4°F) measured at least one time on 2 successive days, and while febrile, subjects had to have at least two of the signs or symptoms listed: nausea, vomiting, headache, eye pain, muscle ache, joint ache, abdominal pain, sore throat, or rash. Any suspected case underwent additional evaluation for dengue (history, focused physical examination, CBC, ALT, AST, and DENV viremia). In addition to passive surveillance, subjects were questioned and examined for signs and symptoms on the day of each TDEN vaccine/placebo administration and weekly within 1 month after each vaccination.

A confirmed dengue case was a suspected case that met the additional following criteria: (1) positive for dengue viremia (DENV RNA by reverse-transcription quantitative polymerase chain reaction [RT-qPCR]) and (2) at least one of the following clinical laboratory abnormalities: absolute neutrophil count (ANC) ≤ 1,000 cells/μL, AST or ALT > 2.5 times the upper limit of normal, hemoconcentration during fever or 1 day after defervescence (peak hematocrit ≥ 1.2 times baseline), or thrombocytopenia < 100,000 cells/mL.

A tourniquet test was performed at the first visit for baseline assessment and two times within the first 2 weeks after the first dose to assess for altered dermal vascular permeability. A positive tourniquet test was defined as 20 or more petechiae within a 2.5-cm square.

CBC, ANC, hematocrit, ALT, and AST were measured on blood samples taken on the day of each vaccine/placebo administration, two times within the first 2 weeks after each dose (one time on day 2, 5, 8, or 12 and again on day 5, 12, or 14 as determined by random allocation), and 30 days after each dose. Laboratory alert values included platelet count < 100,000 cells/mm^3^, ANC < 1,000 cells/mm^3^, and ALT or AST > 2.5 times the upper limit of normal.

We also evaluated subjects for dengue viremia two times within the first 2 weeks after each vaccine/placebo dose (one time on day 2, 5, 8, or 12 and again on day 5, 12, or 14 as determined by random allocation), on day 30 after each dose, and whenever dengue was suspected. Detection of DENV RNA was measured using RT-qPCR based on an assay protocol modified from Sadon and others.^13^ Any positive serum samples underwent a partial genomic sequence (E gene) analysis performed to characterize the DENV type and determine its origin (vaccine virus or wild-type virus).

### Evaluation of immune response.

To characterize TDEN vaccine immunogenicity and pre-existing dengue antibody status, DENV neutralizing antibodies were measured on the day of each vaccination and 1 and 3 months after each dose. Antibodies to each DENV type were measured at the Translational Medicine Branch—Pilot Bioproduction Facility, WRAIR using a quantitative microneutralization assay (MN50) performed in Vero cells as previously described.^10^ Seropositivity was defined as a titer with ≥ 1:10 dilution.

This new MN50, also used in the previous study among US subjects,^10^ was shown to be specific and sensitive for the detection of anti-DENV neutralizing antibodies (e.g., limit of the blank < 1:3.3, limit of detection < 1:7, and limit of quantification ≤ 1:10 for all four serotypes). The precision of the assay was estimated to range from 39% to 59%, depending on serotype.

JEV neutralizing antibodies were measured on blood samples collected at day 0 using a plaque reduction neutralization test (PRNT_50_) with fourfold dilutions of sera. The assay was performed at the US Army Medical Component–Armed Forces Research Institute of Medical Sciences (AFRIMS) in Bangkok, Thailand using the attenuated JEV SA-14-14-2 strain. Seropositivity to JEV was defined as a titer ≥ 1:10 dilution.

### Statistical analysis.

#### Safety.

The safety analysis was performed on all enrolled subjects who received at least one dose (total vaccinated cohort). Descriptive statistics were performed. The overall percentages of subjects reporting an AE after vaccine administration (21 days for solicited AEs after each vaccination and 31 days after any dose for spontaneously reported unsolicited symptoms) were tabulated with exact 95% confidence intervals (95% CIs) by type of AE and intensity (any grade and grade 3). All SAEs occurring during the study were listed for each treatment group. The proportion of subjects with abnormal physical examination findings detected up to 31 days after each TDEN vaccine or placebo administration was tabulated with exact 95% CI.

#### Viremia.

The limit of detection (LOD) was used as the cutoff to determine the presence of viremia. A subject was positive for viremia if the assay value was greater than or equal to the LOD, negative if the assay value was zero, and indeterminate if the assay value was greater than zero and less than the LOD. The LODs expressed in log genome equivalents (GEQs) per milliliter were 2.7 log GEQ/mL for DENV-1, DENV-2, and DENV-3 and 3.4 log GEQ/mL for DENV-4.

#### Immunogenicity.

The analysis of immunogenicity was based on the according-to-protocol (ATP) cohort (i.e., subjects who met all eligibility criteria, who complied with protocol procedures, who had no elimination criteria during the study, and for whom data were available for at least one immunogenicity endpoint). Seropositivity rates and the proportion of subjects with a tetravalent response were reported by group with exact 95% CIs. The proportion of subjects with a tetravalent response was defined, at each time point, as the percentage of subjects with antibody titers ≥ 1:10 for all four DENV types. Geometric mean titers (GMTs), reported with 95% CIs, were computed by taking the antilog of the mean of the log-transformed titers, with a value of 5 (one-half the cutoff) given to titers < 1:10 and a value of 2,430 given to titers > 1:2,430. Analyses were performed based on pre-vaccination flavivirus priming status. A flavivirus-primed subject was defined as having a DENV neutralizing antibody titer ≥ 1:10 to at least one DENV type or a JEV neutralizing antibody titer ≥ 1:10.

## Results

### Study population.

**Figure 1.**
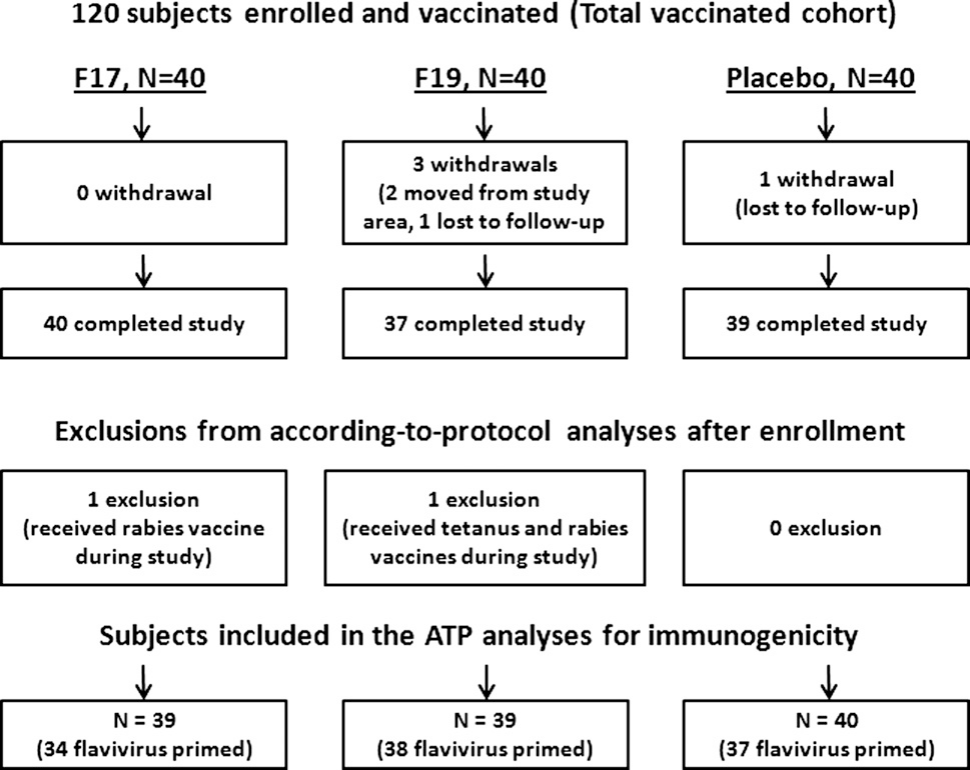
Subject disposition. *N* = number of subjects; ATP = according to protocol cohort.

In total, 120 subjects (40 subjects per group) were enrolled, and 116 subjects (97%) completed the study. Two subjects were withdrawn from the study because of a move from the area, and two subjects were lost to follow-up. None of the withdrawals were related to an AE. Figure 1 shows subject disposition. All enrolled subjects were Thai. Their mean age was 21.6 years (ranging from 20 to 24 years), and 56.7% were female.

Among the total vaccinated cohort, 92.5% of the subjects were primed to either a DENV or JEV before receiving their first dose of vaccine/placebo (87.5% and 97.5% in the two vaccine groups [F17 and F19, respectively] and 92.5% in the placebo group); 40 of 120 subjects were primed to JEV: of these subjects, 87.5% were primed to at least one DENV type, and 82.5% (75–93.3% per group) had neutralizing antibody against all four DENV types. Of 78 subjects in the total vaccinated cohort who were not primed to JEV, 88.5% were primed to at least one DENV type, and 73.1% were primed to all four DENV types (50–88.5% per group). The pre-vaccination priming status to JEV was not determined for 2 of 120 subjects enrolled. These two subjects were seropositive for all four DENV types.

### Safety results.

#### Clinical safety of the vaccine.

Solicited injection site reactions reported per subject over both doses and after each dose separately are presented in Table 2. All solicited injection site reactions were assumed to be causally related to vaccination. After the first dose, injection site pain was reported by 35% of the subjects in the F17 group and 15.4% and 15% in the F19 and placebo groups, respectively. Among the three groups, redness was reported by 20–30%, and swelling was reported by 5–12.5% of the subjects after the first dose. There were no grade 3 injection site reactions reported in any group after either dose. Injection site reaction reporting did not increase with the second dose.

The incidence of solicited general AEs reported per subject over both doses is presented in Table 3. Fatigue was the most frequently reported general symptom (reported by 72.5% [95% CI = 56.1–85.4%] of subjects in the F17 group, 43.6% [95% CI = 27.8–60.4%] of subjects in the F19 group, and 42.5% [95% CI = 27.0–59.1%] of subjects in the placebo group) followed by headache (reported by 70% of subjects in the F17 group, 25.6% of subjects in the F19 group, and 42.5% of subjects in the placebo group). There was a low incidence of grade 3 general AEs reported after both doses, with seven grade 3 AEs reported in the F17 group (rash [*N* = 2], fatigue, headache, nausea, fever, and pruritus), one grade 3 AE reported in the placebo group (fever), and no AEs reported in the F19 group. The incidence of fatigue, headache, muscle aches, nausea, pain behind the eyes, and rash was similar between the F19 group and placebo group, but based on this small number of subjects, it seemed to be higher in the F17 group overall. Vomiting was the least reported solicited AE in all groups.

The percentage of subjects with at least one unsolicited AE reported in any group ranged from 52.5% to 57.5%. The most frequently reported unsolicited symptoms in F17, F19, and placebo groups were dysmenorrhoea (17.5%, 7.5%, and 5.0%, respectively), nasopharyngitis (15.0%, 10.0%, and 5.0%, respectively), pharyngitis (10%, 10%, and 7.5%, respectively), and upper respiratory tract infection (12.5%, 0.0%, and 10.0%, respectively). Grade 3 unsolicited symptoms were rare; one grade 3 unsolicited AE was reported in the F19 group (animal bite), and two grade 3 unsolicited AEs were reported in the placebo group (abdominal pain and appendicitis) for two different subjects. There were no grade 3 unsolicited symptoms reported after either F17 dose.

A laboratory alert value (> 2.5 times the upper limit of normal for AST) was reported for one subject (F19 group). This subject had an AST of 87 U/L approximately 2 weeks after dose 2, which returned to normal by the next visit (at 1 month after dose 2); it was not considered to be related to study vaccination according to the investigators. No viremia or any unsolicited symptom was reported at the time of elevated AST. Overall, ALT and AST levels seemed similar among the three groups. Elevated ALT levels that were normal at baseline were reported (by a maximum of one subject per group at any given time point), and elevated AST levels that were normal at baseline were reported by a maximum of two subjects per group at any given time point.

None of the subjects with normal hematocrit levels at baseline had elevated levels after vaccinations.

All of the TDEN vaccine recipients with normal neutrophil and platelet counts at baseline also had normal levels throughout the study. One subject in the placebo group with normal levels at baseline had a low neutrophil count within the first 2 weeks after dose 1, but none had a low platelet count.

There were no fatalities during this study. Three SAEs were reported, all of which resolved. None were considered by the investigators to be vaccine-related. Two SAEs were reported in the F19 group: one subject had an abdominal wall hernia beginning 46 days after dose 1 of F19 vaccine, and one subject had an ectopic pregnancy (her pregnancy test at the dose 2 visit was positive, and therefore, vaccination was withheld). The ectopic pregnancy required surgery, leading to termination of the pregnancy. In the placebo group, one subject had appendicitis 11 days after the second placebo dose.

There were no reports of suspected or confirmed dengue after TDEN vaccination. One subject in the placebo group (112) was considered by the investigators to have had a subclinical, wild-type DENV-1 infection.

This subject was flavivirus-naïve at baseline and remained seronegative to all four DENV types up to month 7, at which time DENV-1 viremia was detected (4.9 log GEQ/mL) during the scheduled viremia testing planned in the protocol. Note that, at the time that viremia was detected, the subject had no physical complaints (i.e., no signs or symptoms) or abnormal laboratory tests. At month 9, the subject had a tetravalent dengue response with the following titers (one per dilution): DENV-1, > 2,430; DENV-2, 104; DENV-3, 102; DENV-4, 47.

#### Viremia.

At scheduled visits within 30 days after each dose, dengue viremia was detected in five TDEN recipients. Three subjects had DENV-2 viremia (two subjects in the F17 group and one subject in the F19 group). (1) One subject (37) had 2.8 log GEQ/mL DENV-2 on day 14 after dose 1 of F17, with generalized rash and left cervical lymphadenopathy noted on dengue physical examination. (2) One subject (113) had 3.0 log GEQ/mL on day 12 after dose 2 of F17, with pain behind the eyes for 3 days before viremia was measured but not on day 12. (3) One subject (69) had 5.2 log GEQ/mL on day 14 after dose 1 of F19, with generalized rash and bilateral inguinal lymphadenopathy noted on dengue physical examination. DENV-3 viremia 3.4 log GEQ/mL was detected in one subject (29) in the F19 group on day 14 after dose 1 without any examination findings. DENV-4 viremia 5.1 log GEQ/mL was detected in one subject (65) in the F17 group on day 8 after dose 1, which was accompanied by fatigue, headache, and pain behind the eyes on the same day and 2 days before; fever and muscle aches were reported the day before viremia was measured.

#### Abnormal findings during dengue physical examinations.

The incidences of dengue-focused physical examination findings distinguishable from baseline are shown in Table 4. Lymphadenopathy was the most commonly reported finding, with a similar occurrence among the three groups. Lymphadenopathy was reported more frequently after dose 1 than dose 2 in each group. Most of the dengue physical examination findings were indistinguishable from baseline, including all cases of conjunctival hemorrhage, conjunctival injection, generalized lymphadenopathy, hepatomegaly, mucosal hemorrhage, and splenomegaly.

Generalized rash covering at least 50% of the body (not observed at baseline) was found in 5% of the TDEN vaccine recipients (two subjects in each group) after dose 1 (no findings in placebo group and no findings after dose 2). Rash covering less than 50% of the body (not observed at baseline) occurred only after dose 1 in the vaccine groups and only after dose 2 in the placebo group, with the occurrence ranging from 0% to 5.1% of the subjects. Skin petechial hemorrhage (not observed at baseline) was found in one subject in the F17 group (2.5%) and was not found in the other two groups.

At each time point, most subjects in each vaccine group had a negative tourniquet test after dose 1 (at least 92.3% for any given time point); all subjects in the placebo group had consistently negative tests. Two initially negative subjects in the F19 group had a positive tourniquet test at day 2, and one initially negative subject had a positive test at day 12. In the F17 group, one initially negative subject had a positive test at day 12, and one subject had a positive test at day 14. There did not seem to be an association between the finding of a positive tourniquet test and the occurrence of other dengue symptoms or signs.

#### Cardiac evaluation.

No cardiac events were reported in the vaccine groups, and one suspected case (dizziness/loss of consciousness 1 month after dose 1) was reported in the placebo group that was not present at baseline. The consulting cardiologist was unable to identify a cardiac etiology.

### Immunogenicity results.

#### All subjects.

**Figure 2.**
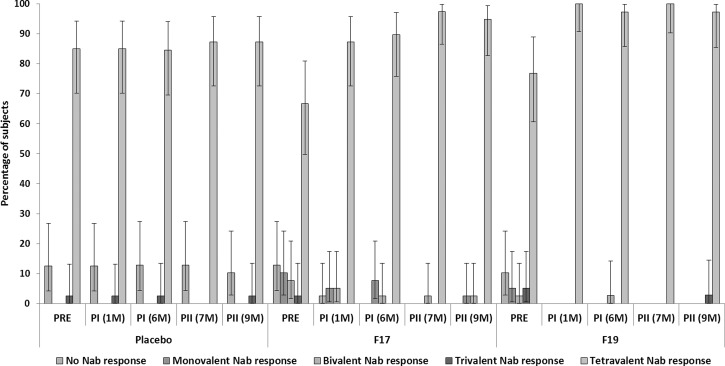
Mono-, bi-, tri-, and tetravalent neutralizing antibody responses to DENV types per group pre- and post-vaccination. M = month of study conduct; Nab = neutralizing antibodies; PI = post-vaccination dose 1; PII = post-vaccination dose 2; PRE = pre-vaccination.

Figure 2 shows the mono-, bi-, tri-, and tetravalent neutralizing antibody responses by group. A tetravalent antibody response was observed in most of the F17 and F19 vaccine recipients after dose 1, which was sustained through the end of the study.

Immunology results are described below based on flavivirus priming status before dose 1.

#### Initially flavivirus-primed subjects.

DENV antibody seropositivity rates and GMTs for primed subjects are presented in Table 5 for the TDEN groups. No differences were observed at the month 1 and 3 time points after each dose (only month 1 time point is shown in Table 5).

GMTs were similar between the two vaccine groups. For all four DENV types, there was an increase in titers 1 month after dose 1 (from pre-vaccination levels), but GMTs remained fairly consistent from 1 month after dose 1 to 1 and 3 months after the second dose.

Seropositivity rates to at least one DENV for initially flavivirus-primed subjects in the TDEN groups ranged from 82.4% to 89.5% at pre-vaccination. All flavivirus-primed subjects (100%) in the F19 group and 97.1% of flavivirus-primed subjects in the F17 group were seropositive for antibodies against all four DENV types 1 month after each vaccine dose: at pre-vaccination, 76.5% and 78.9% of subjects in F17 and F19 groups, respectively, were primed to all four DENV types. Seropositivity rates remained the same when measured again 2 months after dose 2.

Six subjects in either vaccine group were classified as having pre-vaccination monovalent DENV priming (i.e., antibody titers to only one DENV type); five subjects acquired tetravalent seroconversion 1 month after dose 1, and five subjects had tetravalent seropositivity through months 7 and 9.

Of 63 subjects in both TDEN vaccine groups who were primed to more than one DENV type before dose 1, 56 subjects had antibodies against all four DENV types at baseline. All seven subjects who did not have tetravalent antibodies before vaccination acquired tetravalent antibodies 1 month after dose 1.

Among the primed subjects in both groups, post-vaccination GMTs to DENV-2 and DENV-1 seemed slightly higher than those GMTs observed for DENV-3 and DENV-4.

#### Initially flavivirus-unprimed subjects.

Nine subjects were unprimed to flavivirus (*N* = 5, 1, and 3 in F17, F19, and placebo groups, respectively) before vaccination (Table 6). Among five unprimed subjects in the F17 group, one subject had a tetravalent response 1 month after a single dose, all subjects were seropositive for antibodies to each DENV type 1 month after the second dose, and four subjects were seropositive to each DENV type 3 months after the second dose. The single unprimed subject in the F19 group had a tetravalent response 1 month after each dose. At 3 months after dose 2 across all four DENV types, antibody titers decreased or remained unchanged (data not shown).

Among these six initially unprimed subjects in both F17 and F19 groups, five subjects had an increase in DENV-1 and DENV-3 from dose 1 to dose 2, four subjects had an increase in DENV-2, and two subjects had an increase in DENV-4 (and two other subjects had high DENV-4 titers [2,430] at both time points).

Among three placebo recipients who were unprimed to flavivirus at baseline, two subjects had no measurable DENV antibody at any of the post-vaccination time points as expected. One subject did have antibodies to all four DENV types at 3 months after dose 2; however, as previously mentioned, this subject was considered by the investigators to have had a subclinical, wild-type DENV-1 infection.

## Discussion

Results from this phase II, double blind, placebo, controlled clinical trial of the WRAIR GSK live-attenuated, tetravalent TDEN vaccine candidate showed that both formulations of the vaccine candidate had a clinically acceptable safety and immunogenicity profile in a small number of highly flavivirus-primed Thai adults.

The F19 formulation was created in an attempt to decrease reactogenicity and improve the balance and potency of the overall immune response. In this trial, it seems that the F19 formulation had a more favorable profile than the F17 formulation in terms of injection site pain and general symptoms; however, no comparative conclusions can be drawn in this small study. The incidence of grade 3 solicited symptoms was rare, and no SAEs related to vaccination were reported during the study. There were no reports of suspected or confirmed dengue after TDEN vaccination and no clinical or clinical laboratory safety signals. The balance of the immune response was similar between F17 and F19 groups. As previously reported,^10^ the DENV-4 strain in the candidate vaccine has the fewest cell culture passages among the four strains, and the F19 formulation was planned to have 10-fold less DENV-4 than the F17 formulation (the measured reduction was fourfold, but the test error was ± twofold).

The safety results from this study were not substantially different from those results reported when the same vaccine candidates were administered on a 0- and 6-month schedule to predominantly unprimed US adults.^10^ Dengue physical examination findings were reported similarly among the two studies, and in both studies, the incidence of viremia was low, no AEs of cardiac origin were reported after vaccination with either candidate vaccine, and there were no unsolicited symptoms or laboratory tests that triggered a safety issue. The one exception was solicited symptom reporting for the F17 and F19 groups, which in general, tended to be somewhat higher in this study. In particular, injection site pain and fever were reported at least two times as frequently in this Thai study than in the US study. Symptom reporting in the placebo group was not noticeably different between the two studies.

Viremia was measured two times in the first 2 weeks after each vaccination and again 30 days after vaccination. It was also measured any time that a subject reported fever to the investigator. The frequency of the testing was intended to capture any responses to vaccination during the period when replication of the vaccine viruses was expected to be at a maximum. Monotypic viremia (DENV-2, DENV-3, and DENV-4), ranging from 2.8 to 5.2 log GEQ/mL, was detected in 5 of 80 TDEN recipients with no resulting illness. Notably, four of five viremic subjects had pre-vaccination (baseline) neutralizing antibodies to the DENV type that caused viremia that were undetectable (< 1:10): DENV-2 viremia occurred in three subjects who had no baseline DENV antibody (*N* = 2) or had DENV-1 or DENV-4 antibodies only (*N* = 1), and DENV-3 viremia occurred in one subject with baseline DENV-2 antibody only. Exceptionally, DENV-4 viremia occurred in one subject with low-level baseline antibody to DENV-4 (1:11) and higher levels of antibodies to the other three DENV types (range of 1:49 to 1:397). Because viremia testing was conducted only two times over a 2-week period, it is possible that an association between vaccine dengue viremia and fever may have gone undetected. Nevertheless, these preliminary data support the safety of administering the TDEN vaccine candidate to individuals with a diverse range of pre-vaccination antibodies.

There were no cases of dengue detected (neither dengue caused by vaccine nor wild-type virus) in either vaccine group. However, one subject in the placebo group was considered by the investigators to have had a subclinical (asymptomatic and no hematologic and serum chemistry abnormalities), wild-type DENV-1 infection during the study. This subject was flavivirus-naïve at baseline and remained seronegative to all four DENV types up to month 7. DENV-1 viremia (4.9 log GEQ/mL) was detected at the scheduled day 30 post-vaccination time point, at which time the subject was asymptomatic. Two months later, this subject had a tetravalent dengue response. Such documented asymptomatic viremia in humans from naturally acquired dengue infection is infrequently reported in the literature.^14^

The first documentation of asymptomatic viremia in naturally acquired human dengue infection was reported in Indonesia.^15^ In adult populations, primary dengue infections are usually symptomatic, unlike in children.^16^,^17^

Lymphadenopathy was the most commonly reported finding at dengue physical examination: most cases were indistinguishable from baseline. There were no findings of conjunctival or mucosal hemorrhage, conjunctival injection, or splenomegaly distinguishable from baseline in any group. One case of skin hemorrhage distinguishable from baseline was reported in the F17 group. The incidence of rash distinguishable from baseline was rare and similar among all groups.

A tourniquet test is often used as a tool in Asian children in an attempt to distinguish dengue from other febrile illnesses. In our study, there did not seem to be an association between a positive tourniquet test and other dengue symptoms. We found that the use of this test did not enhance the value of the routine clinical evaluations of vaccine reactogenicity directed at finding signs related to dengue virus infection during the period after each vaccine dose. The number of baseline negative subjects with positive tests post-vaccination was rare and similar among groups. A recent study of adults in the Americas showed that a positive tourniquet test alone was specific but not sensitive in distinguishing dengue from other acute febrile illness; however, the absence of leucopenia combined with a negative tourniquet test may be useful to rule out dengue.^18^

Knowledge of baseline priming status is essential in assessing vaccine immune responses.^19^ Data were analyzed based on priming status to any of the DENV types and JEV, which is coendemic in Thailand. We found that 92.5% of subjects in the total vaccinated cohort were already primed to either a DENV or JEV. One dose of either candidate vaccine was sufficient to induce an antibody response for flavivirus-primed subjects, which was evidenced by a substantial increase in GMTs from pre-vaccination to 1 month after dose 1 vaccination, despite high levels of pre-existing antibody titers to one or more DENV types. However, among six unprimed subjects in the vaccine groups, two doses were necessary to elicit a tetravalent immune response for four of these subjects. There was a substantial increase from dose 1 to dose 2 in these subjects that may indicate that the second dose expanded and broadened the immune response induced by the first dose. These variations in responses were expected and have been reported elsewhere with other investigational dengue vaccines given in endemic and non-endemic areas.^10^,^20^

A majority of unprimed subjects elicited neutralizing antibody responses to all four DENV types, which were consistent with responses reported in unprimed subjects in the US study of the same candidate vaccines.^10^ The TDEN vaccine candidate (both F17 and F19) promoted the small number of subjects with a monovalent pre-vaccination neutralizing antibody profile (a potential risk factor for enhanced disease) to a trivalent or tetravalent profile. This result suggests that people who may be at increased risk for a more severe dengue infection may derive benefit from the vaccine, because risk of a more severe infection might be reduced. If this response were to be sustained beyond the 3-month follow-up period, vaccination could be expected to be beneficial to this population. The value of eliciting a vaccine response that promotes a subject from a monotypic neutralizing antibody response to a multitypic response is uncertain, although it could potentially be beneficial, because epidemiologic studies have shown that having monotypic neutralizing antibody is a risk factor for dengue hemorrhagic fever.^21^,^22^

In our study, the immune responses to DENV-2 and DENV-1 types seemed slightly superior to the immune responses observed for the DENV-3 and DENV-4 types among the flavivirus-primed subjects. In the previous trial of the same vaccine formulations, this trend was observed among flavivirus-unprimed subjects.^10^ In previous trials with this dengue vaccine,^8^,^9^ the four serotypes in the tetravalent vaccine differed in their ability to elicit an immune response. We acknowledge that, because of the presence of cross-reactive neutralizing epitopes on DENV surface proteins, the appearance of antibody to a DENV type does not imply that vaccine virus of that type replicated sufficiently to elicit a virus-specific immune response.

## Conclusion

The WRAIR GSK TDEN vaccine candidate has an acceptable reactogenicity and safety profile in flavivirus-primed healthy young adults, and it is immunogenic, eliciting responses that have the potential to confer clinical benefit to all DENV types. A study of this candidate tetravalent vaccine in a broad age range with adequate sample size would be needed to further assess safety and immunogenicity and evaluate whether F17 or F19 is suitable for additional development.

## Figures and Tables

**Table 1:** Dengue vaccine formulations and placebo

Dengue vaccine formulations in vitro potency (log_10_ focus forming units per milliliter) of each DENV strain
Dengue F17
DENV-1: 4.9 log_10_ FFU/mL
DENV-2: 5.3 log_10_ FFU/mL
DENV-3: 4.7 log_10_ FFU/mL
DENV-4: 5.0 log_10_ FFU/mL
Dengue F19
DENV-1: 4.9 log_10_ FFU/mL
DENV-2: 5.2 log_10_ FFU/mL
DENV-3: 4.6 log_10_ FFU/mL
DENV-4: 4.4 log_10_ FFU/mL
Placebo
A sterile solution of EMEM with the same virus stabilizer contained in the vaccine (vegetable-derived carbohydrates and amino acids); the placebo was identical in appearance to the dengue vaccine
Viral origin and manufacturer
DENV-1: 45AZ5, PDK 27
DENV-2: S16803, PDK 50
DENV-3: CH53489, PDK 20
DENV-4: 341750, PDK 6
All vaccine lots and placebo were produced at the US Army WRAIR Pilot Bioproduction Facility in Silver Spring, MD

FFU = focus forming units.

**Table 2:** Number and percentage of subjects reporting injection site reactions during the 21-day follow-up period post-vaccination (total vaccinated cohort)

Injection site		F17 (*N* = 40)			F19 (*N* = 39)			Placebo (*N* = 40)		
Reactions	Type	*n*	%	95% CI	*n*	%	95% CI	*n*	%	95% CI
Both doses/subject										
Pain	All	19	47.5	31.5–63.9	10	25.6	13.0–42.1	10	25.0	12.7–41.2
Redness	All	16	40.0	24.9–56.7	15	38.5	23.4–55.4	9	22.5	10.8–38.5
Swelling	All	9	22.5	10.8–38.5	6	15.4	5.9–30.5	5	12.5	4.2–26.8
Dose 1										
Pain	All	14	35.0	20.6–51.7	6	15.4	5.9–30.5	6	15.0	5.7–29.8
Redness	All	12	30.0	16.6–46.5	11	28.2	15.0–44.9	8	20.0	9.1–35.6
Swelling	All	5	12.5	4.2–26.8	4	10.3	2.9–24.2	2	5.0	0.6–16.9
Dose 2										
Pain	All	13	33.3	19.1–50.2	7	20.0	8.4–36.9	6	15.4	5.9–30.5
Redness	All	13	33.3	19.1–50.2	8	22.9	10.4–40.1	6	15.4	5.9–30.5
Swelling	All	7	17.9	7.5–33.5	4	11.4	3.2–26.7	5	12.8	4.3–27.4

All = includes all grades (1–3) reported; *N* = number of subjects with at least one documented dose; *n*/% = number/percentage of subjects reporting the symptom at least one time; No grade 3 injection site reaction reported; 95% CI = exact 95% confidence interval.

**Table 3:** Number and percentage of subjects reporting solicited general AEs during the 21-day follow-up period post-vaccination (total vaccinated cohort)

	Type	F17 (*N* = 40)			F19 (*N* = 39)			Placebo (*N* = 40)		
		*n*	%	95% CI	*n*	%	95% CI	*n*	%	95% CI
Fatigue	All	29	72.5	56.1–85.4	17	43.6	27.8–60.4	17	42.5	27.0–59.1
Fatigue	Grade 3	1	2.5	0.1–13.2	NR			NR		
Fever	All	18	45.0	29.3–61.5	15	38.5	23.4–55.4	13	32.5	18.6–49.1
Fever	Grade 3	1	2.5	0.1–13.2	NR			1	2.5	0.1–13.2
Headache	All	28	70.0	53.5–83.4	10	25.6	13.0–42.1	17	42.5	27.0–59.1
Headache	Grade 3	1	2.5	0.1–13.2	NR			NR
Muscle aches	All	19	47.5	31.5–63.9	9	23.1	11.1–39.3	10	25.0	12.7–41.2
Nausea	All	7	17.5	7.3–32.8	2	5.1	0.6–17.3	4	10.0	2.8–23.7
Nausea	Grade 3	1	2.5	0.1–13.2	NR			NR
Pain behind eyes	All	16	40.0	24.9–56.7	7	17.9	7.5–33.5	9	22.5	10.8–38.5
Abdominal pain	All	4	10.0	2.8–23.7	3	7.7	1.6–20.9	4	10.0	2.8–23.7
Arthralgia	All	6	15.0	5.7–29.8	3	7.7	1.6–20.9	1	2.5	0.1–13.2
Photophobia	All	4	10.0	2.8–23.7	1	2.6	0.1–13.5	3	7.5	1.6–20.4
Pruritus	All	9	22.5	10.8–38.5	7	17.9	7.5–33.5	7	17.5	7.3–32.8
Pruritus	Grade 3	1	2.5	0.1–13.2	NR			NR
Rash	All	9	22.5	10.8–38.5	3	7.7	1.6–20.9	4	10.0	2.8–23.7
Rash	Grade 3	2	5.0	0.6–16.9	NR			NR
Vomiting	All	1	2.5	0.1–13.2	1	2.6	0.1–13.5	1	2.5	0.1–13.2

No grade 3 symptoms were reported for abdominal pain, arthralgia, muscle aches, pain behind eyes, photophobia, and vomiting. All = includes all grades (1–3) reported after both doses; fever = oral temperature ≥ 37.5°C; grade 3 fever = oral temperature > 39.0°C; *N* = number of subjects with at least one documented dose; *n*/% = number/percentage of subjects reporting the symptom at least one time; NR = none reported; Gr 3 = grade 3; 95% CI = exact 95% confidence interval.

**Table 4:** Incidence of dengue physical examination findings during the 31-day follow-up period (total vaccinated cohort)

Symptom	F17			F19			Placebo		
	*n*	%	95% CI	*n*	%	95% CI	*n*	%	95% CI
Dose 1	*N* = 40			*N* = 40			*N* = 40		
Lymphadenopathy	8	20.0	9.1–35.6	10	25.0	12.7–41.2	10	25.0	12.7–41.2
Rash < 50% of the body	2	5.0	0.6–16.9	2	5.0	0.6–16.9	NR
Rash ≥ 50% of the body	2	5.0	0.6–16.9	2	5.0	0.6–16.9	NR
Skin hemorrhage	1	2.5	0.1–13.2	NR			NR
Dose 2	*N* = 40			*N* = 38			*N* = 39		
Lymphadenopathy	3	7.5	1.6–20.4	2	5.3	0.6–17.7	2	5.1	0.6–17.3
Rash < 50% of the body	NR			NR			2	5.1	0.6–17.3
Rash ≥ 50% of the body	NR			NR			NR

During focused examinations, none of the study subjects had a positive finding for splenomegaly, conjunctival hemorrhage, conjunctival injection, hepatomegaly, or mucosal hemorrhage. Skin hemorrhage was only reported after dose 1. *N* = number of subjects with available results; *n*/% = number/percentage of subjects reporting the particular symptom; NR = none reported; 95% CI = exact 95% confidence interval; LL = lower limit; UL = upper limit.

**Table 5:** Seropositivity rates and GMTs to each DENV type 1 month after each dose in flavivirus-primed subjects administered TDEN vaccines (according to the protocol cohort)

DENV type	Pre-vaccination			Post-dose 1 (month 1)			Post-dose 2 (month 7)			Post-dose 2 (month 9)		
	GMT (95% CI)	≥ 1:10		GMT (95% CI)	≥ 1:10		GMT (95% CI)	≥ 1:10		GMT (95% CI)	≥ 1:10	
		*n*	%		*n*	%		*n*	%		*n*	%
F17 group	*N* = 34			*N* = 34			*N* = 34			*N* = 34		
1	289.9 (133.5–629.2)	30	88.2	1,173.4 (741.9–1,855.8)	33	97.1	1,041.2 (665.2–1,629.8)	34	100	916.0 (554.0–1,514.5)	33	97.1
2	368.9 (184.4–738.0)	30	88.2	1,292.3 (951.8–1,754.7)	34	100	1,221.2 (932.6–1,599.1)	34	100	1,362.0 (1,030.1–1,800.9)	34	100
3	186.9 (83.7–417.3)	28	82.4	793.5 (469.4–1,341.6)	33	97.1	602.5 (338.1–1,073.9)	33	97.1	660.6 (375.8–1,161.4)	33	97.1
4	179.9 (88.0–367.9)	29	85.3	819.5 (510.2–1,316.2)	33	97.1	720.5 (426.8–1,216.2)	33	97.1	688.0 (417.1–1,134.7)	33	97.1
F19 group	*N* = 38			*N* = 37			*N* = 35			*N* = 35		
1	463.4 (235.9–910.6)	34	89.5	1,177.9 (828.3–1,675.1)	37	100	1,254.8 (924.2–1,703.5)	35	100	1115.0 (789.8–1,574.1)	35	100
2	309.3 (148.7–643.3)	32	84.2	1,248.2 (895.1–1,740.6)	37	100	1,251.3 (923.1–1,696.0)	35	100	1366.1 (1,047.5–1,781.6)	35	100
3	267.6 (127.3–562.6)	32	84.2	871.3 (552.1–1,375.1)	37	100	878.6 (538.0–1,435.0)	35	100	781.5 (455.9–1,339.8)	34	97.1
4	163.8 (80.1–335.2)	32	84.2	748.9 (506.2–1,107.9)	37	100	671.1 (448.3–1,004.6)	35	100	680.9 (454.0–1,021.4)	35	100

Data collected at month 3 are not presented, because they were similar to data at month 1. *N* = number of subjects with available results at the specified time point; *n* = number of seropositive subjects; pre-vaccination = pre-dose 1; titer ≥ 1:10 = seropositivity.

**Table 6:** Neutralizing antibody titers: flavivirus-unprimed subjects (according to protocol cohort)

PID	Group	DENV	Pre-vac	Post-dose 1 (month 1)	Post-dose 2 (month 7)
90	Placebo	DENV-1	5	5	5
90	Placebo	DENV-2	5	5	5
90	Placebo	DENV-3	5	5	5
90	Placebo	DENV-4	5	5	5
111	Placebo	DENV-1	5	5	5
111	Placebo	DENV-2	5	5	5
111	Placebo	DENV-3	5	5	5
111	Placebo	DENV-4	5	5	5
112	Placebo	DENV-1	5	5	5
112	Placebo	DENV-2	5	5	5
112	Placebo	DENV-3	5	5	5
112	Placebo	DENV-4	5	5	5
36	F17	DENV-1	5	332	191
36	F17	DENV-2	5	1,552	376
36	F17	DENV-3	5	126	35
36	F17	DENV-4	5	2,430	2,430
37	F17	DENV-1	5	5	18
37	F17	DENV-2	5	2,430	441
37	F17	DENV-3	5	5	14
37	F17	DENV-4	5	2,430	516
42	F17	DENV-1	5	5	2,430
42	F17	DENV-2	5	5	47
42	F17	DENV-3	5	5	147
42	F17	DENV-4	5	5	139
46	F17	DENV-1	5	5	2,430
46	F17	DENV-2	5	5	1,439
46	F17	DENV-3	5	5	2,430
46	F17	DENV-4	5	624	2,430
113	F17	DENV-1	5	16	2,340
113	F17	DENV-2	5	5	2,430
113	F17	DENV-3	5	5	572
113	F17	DENV-4	5	2,430	1,706
97	F19	DENV-1	5	131	677
97	F19	DENV-2	5	23	2,430
97	F19	DENV-3	5	19	148
97	F19	DENV-4	5	2,430	2,430

Data collected at months 3 and 9 are not presented, because they were similar to data at months 1 and 7, respectively. PID = patient identification number; pre-vac = before dose 1.
